# Supplementary figures and images for: Mapping protein distribution in the canine photoreceptor sensory cilium and calyceal processes by ultrastructure expansion microscopy
Source: bioRxiv. 2024 Sep 22:2024.06.27.600953. Preprint. [Version 3] doi: 10.1101/2024.06.27.600953 (PMC11230445; doi:10.1101/2024.06.27.600953)

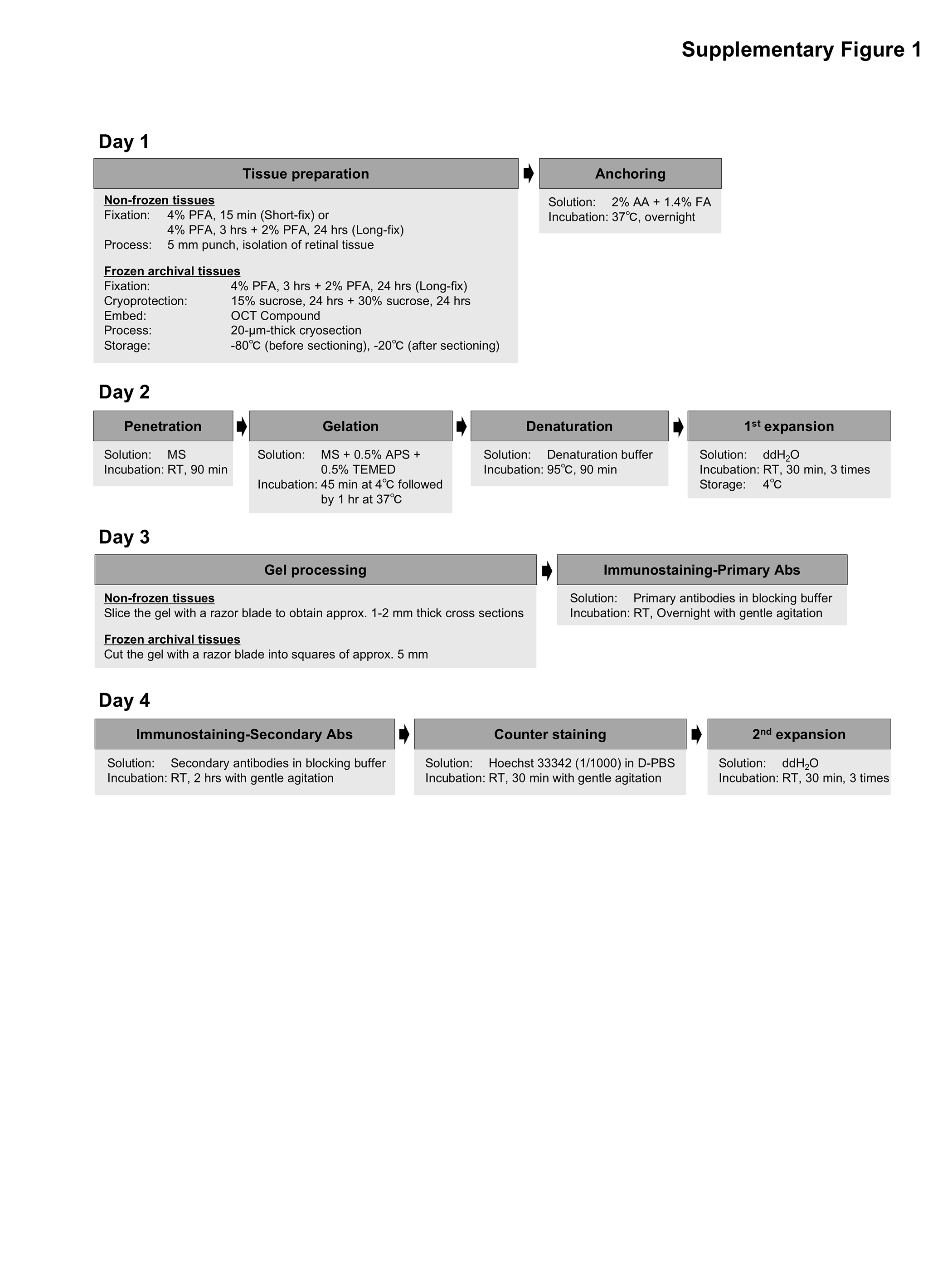

Supplement: Supplement 1 — AA, acrylamide; ASP, ammonium persulfate; ddH2O, distilled deionized water; FA, formaldehyde; MS, monomer solution; OCT, optimal cutting temperature; PFA, paraformaldehyde; RT, room temperature; TEMED, tetramethylethylenediamine. [file media-1.tif]

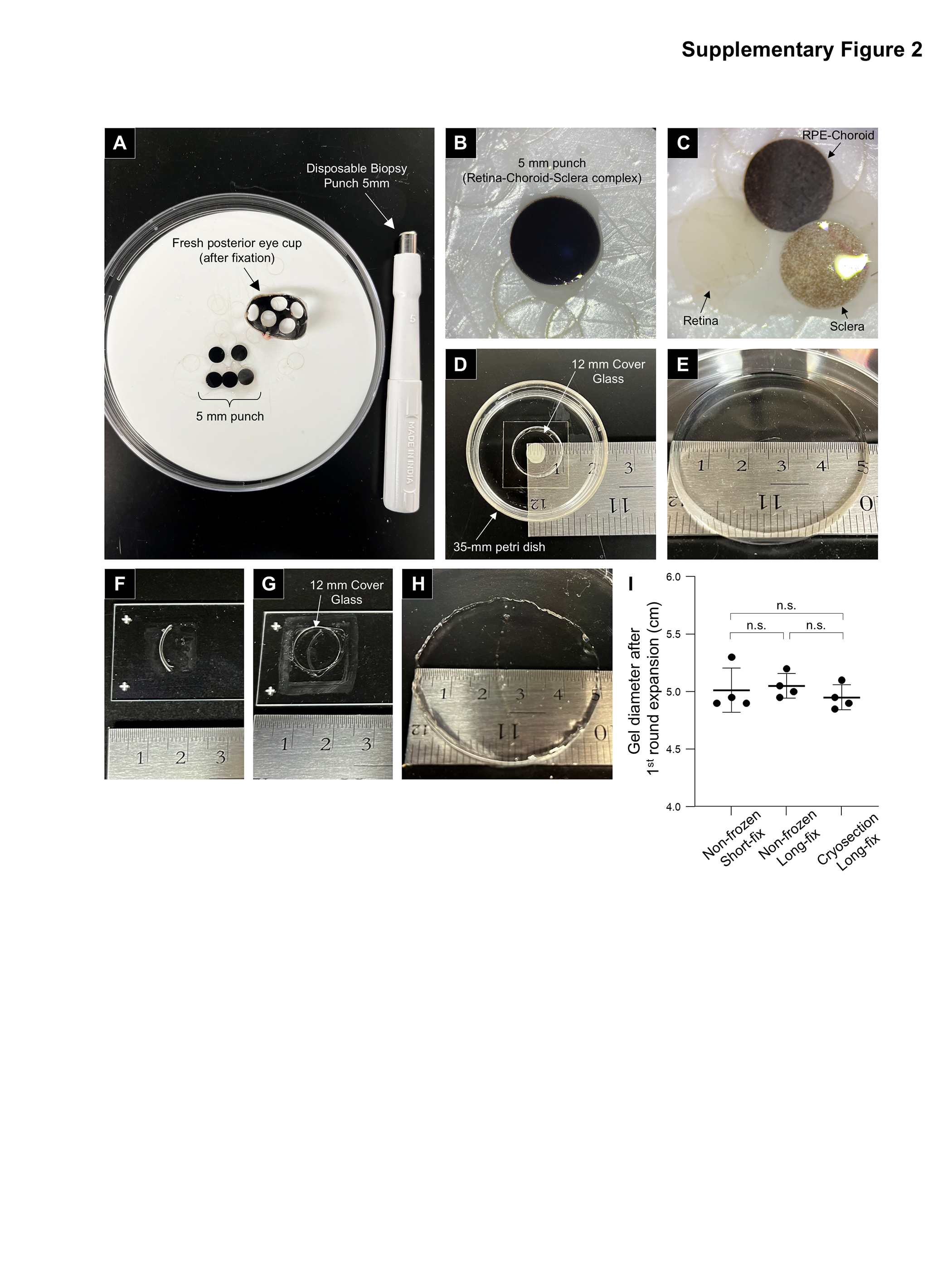

Supplement: Supplement 2 — (A) Collection of 5 mm tissue punches from PFA fixed- non-frozen canine posterior eyecup using a disposable biopsy punch. (B, C) Isolation of retinal tissue from the 5 mm tissue complex (B, before; C, after isolation). (D) Gelation of the 5 mm retinal punch using a 12 mm circular cover glass. (E) First-round expansion of the gel including the non-frozen retinal punch. (F) Twenty-μm-thick canine retinal cryosection. (G) Gelation of the canine retinal cryosection using a 12 mm circular cover glass. (H) First-round expansion of the gel including the retinal section. (I) Comparison of gel diameter after the first-round expansion between different fixation conditions. Sample size: n = 4 individual punches or cryosections from 2–4 eyes per fixation/storage condition. Non-significant (n.s., P > 0.05) by Kruskal-Wallis test with Dunn’s multiple-comparison test. [file media-2.tif]

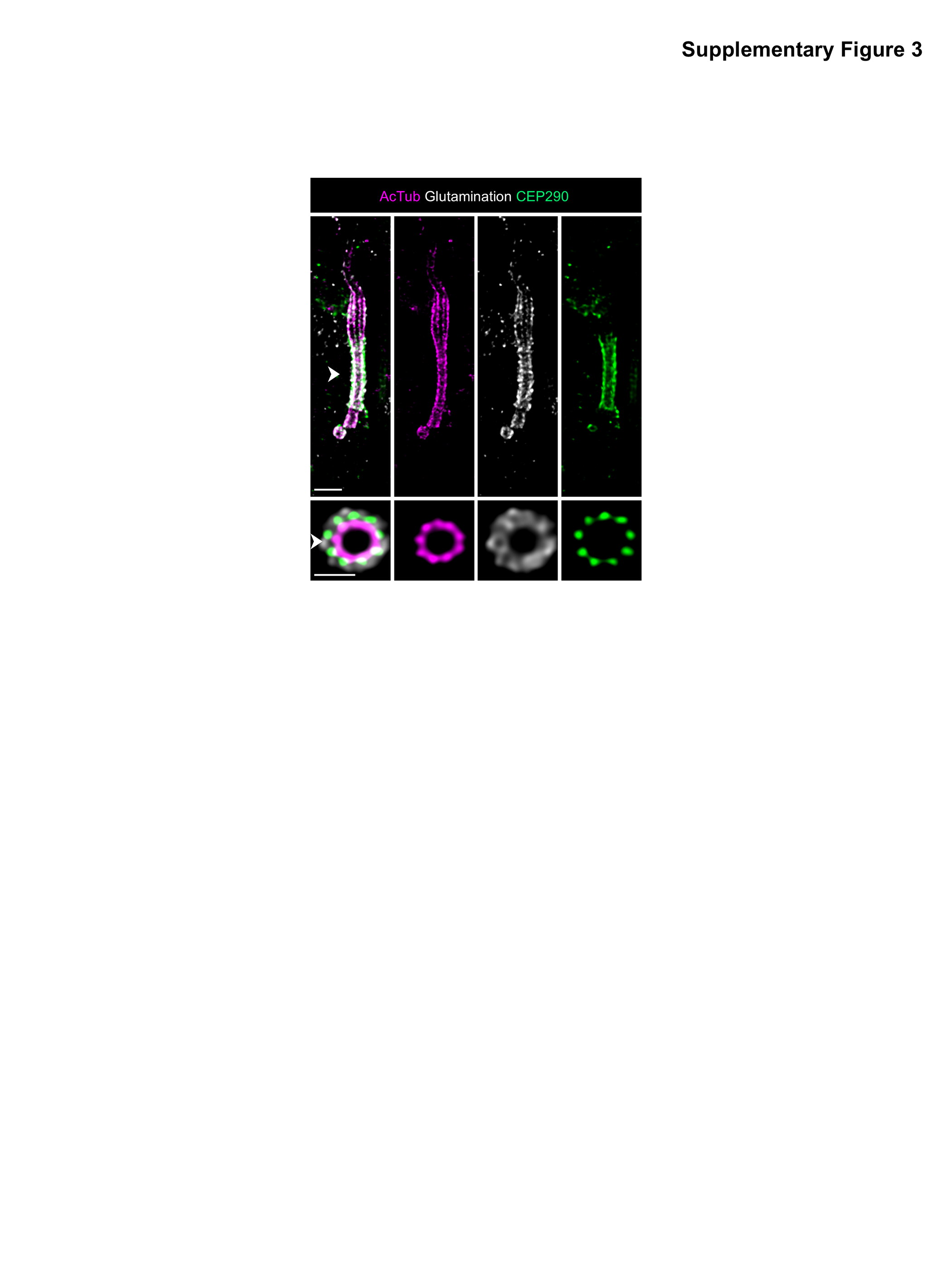

Supplement: Supplement 3 — Confocal images of expanded adult canine PSC stained for AcTub (magenta), glutamination (white), and CEP290 (green). The lower panels show axial views of the CC, indicated by white arrowheads. Scale bars: lateral view = 500 nm; axial view = 200 nm, after correction for expansion factor. Abbreviations: CC, connecting cilium. [file media-3.tif]

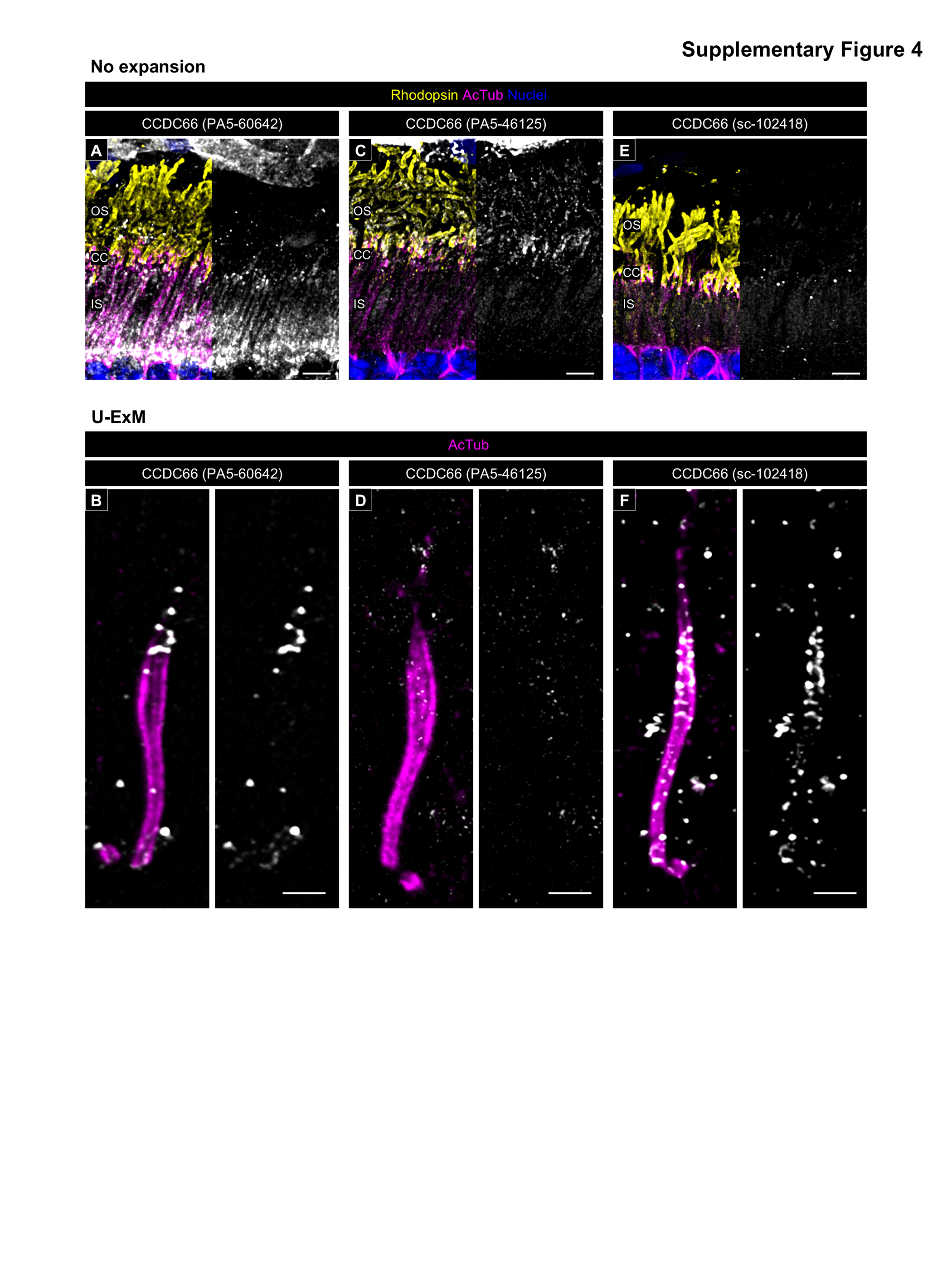

Supplement: Supplement 4 — (A, C, E) Confocal images of non-expanded retinal cryosections stained for rhodopsin (yellow), CCDC66 (white), and AcTub (magenta). Panels (A), (C), and (E) show the results of IHC using three different anti-human CCDC66 antibodies: PA5–60642, PA5–46125, and sc-102418, respectively. Scale bars: 5 μm, without correction for expansion factor. (B, D, F) Expanded normal canine PSC labeled with the PA5–60642 antibody (white, B), PA5–46125 antibody (white, D), and sc-102418 antibody (white, F). The PSC axoneme is visualized by AcTub labeling (magenta). Scale bars: 500 nm, after correction for expansion factor. Abbreviations: CC, connecting cilium; IS, inner segment; OS, outer segment, PSC, photoreceptor sensory cilium. [file media-4.tif]

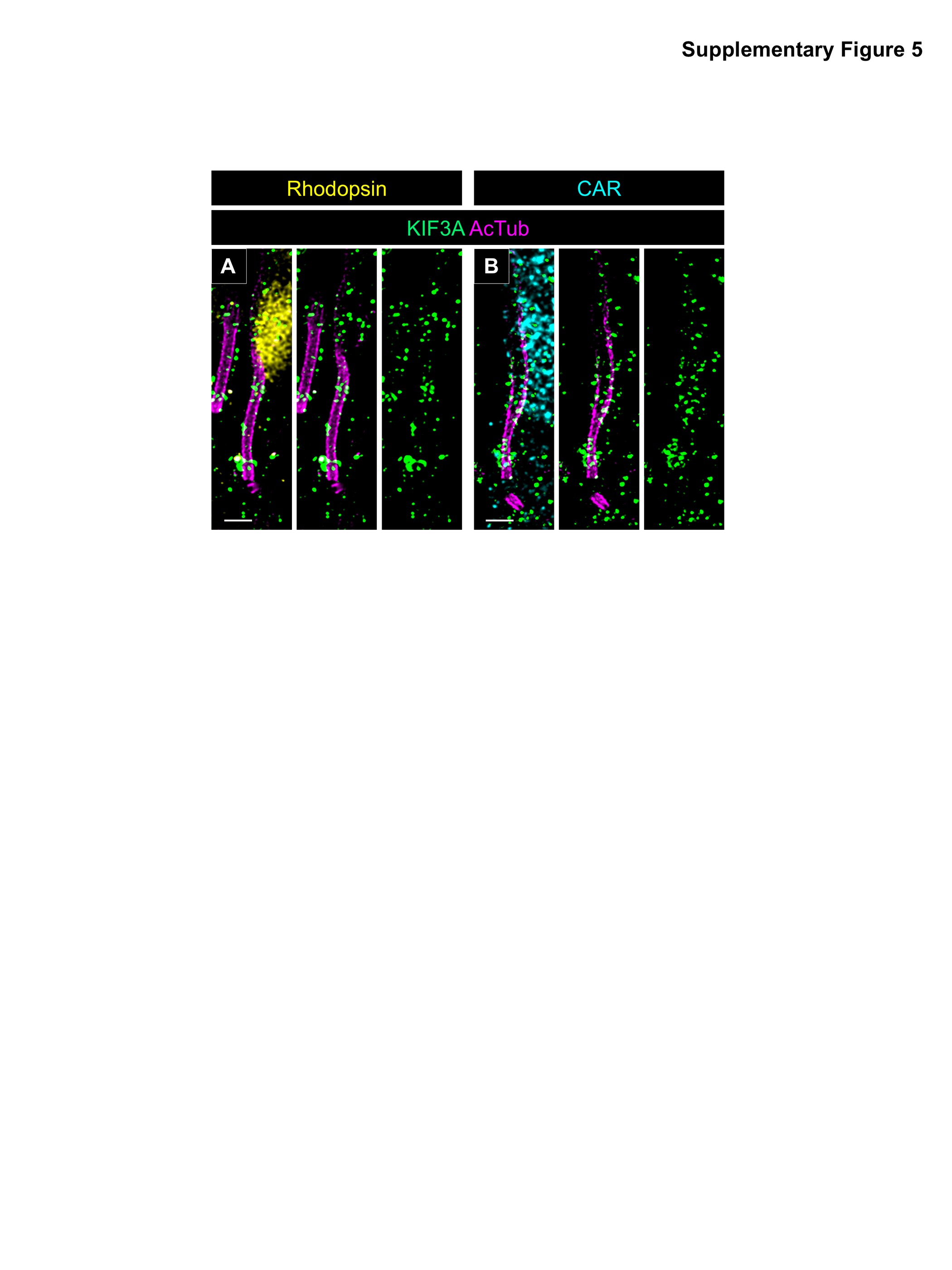

Supplement: Supplement 5 — (A, B) Confocal U-ExM images of normal canine PSC stained for AcTub (magenta) and KIF3A (green) in rod (A) and cone (B) photoreceptors, respectively. KIF3A signal shows a similar localization pattern to IFT57 around the BB and bulge region. Abbreviations: BB, basal body; PSC, photoreceptor sensory cilium. [file media-5.tif]

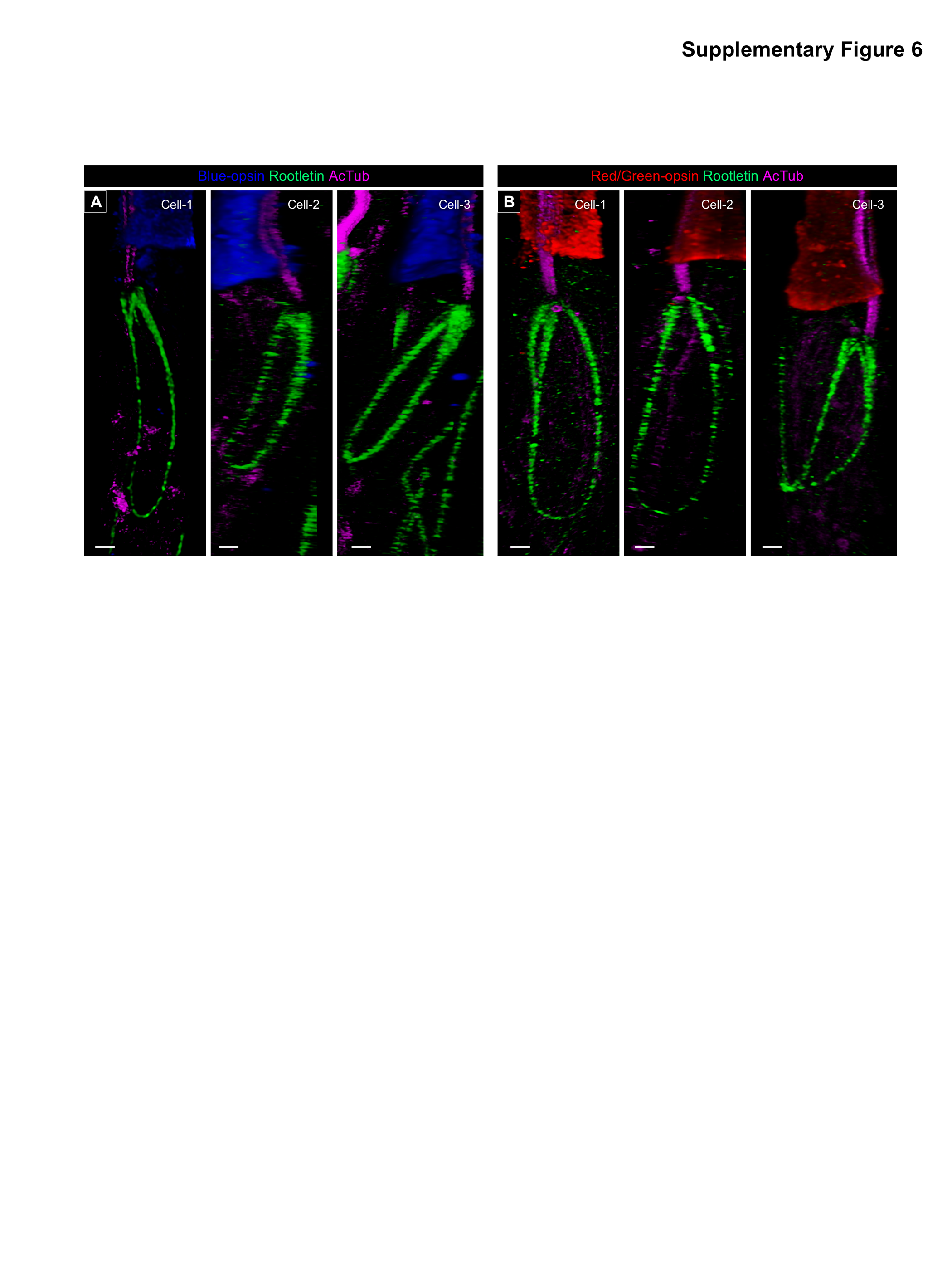

Supplement: Supplement 6 — (A, B) 3D rendering images of U-ExM samples stained for rootletin (green) with blue-opsin (blue, A) or red/green-opsin (red, B). No significant differences are observed in the architecture of the PSC rootlet among the different cone subtypes. Scale bars: 500 nm, after correction for expansion factor. Abbreviation: PSC, photoreceptor sensory cilium. [file media-6.tif]

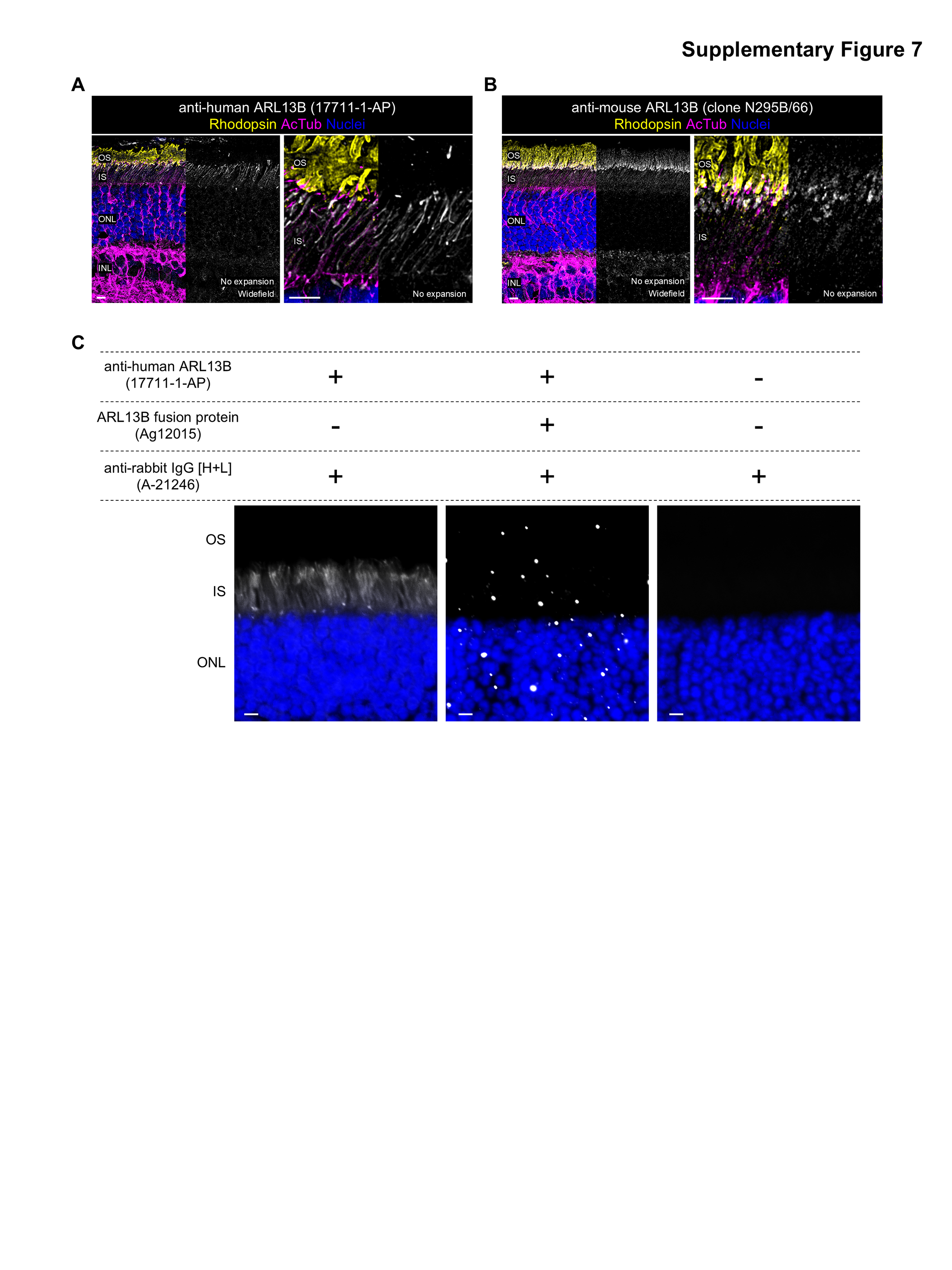

Supplement: Supplement 7 — (A, B) Widefield (left) and high-magnification (right) view of a non-expanded retinal cryosection stained for rhodopsin (yellow), ARL13B (white), and AcTub (magenta). (A) and (B) show the results of IHC using anti-human ARL13B antibody (17711-1-AP) and ant-mouse ARL13B antibody (clone N295B/66), respectively. Consistent with the findings in U-ExM samples, ARL13B labeled with the 17711-1-AP antibody predominantly localized to the IS, whereas the N295B/66 antibody exhibits OS-specific signals in the non-expanded cryosection. (C) Specificity confirmation experiment for the 17711-1-AP antibody. The blocking peptide for the 17711-1-AP antibody completely inhibited its reaction in the IS. Scale bars: 5 μm. Abbreviations: INL, inner nuclear layer; IS, inner segment; ONL, outer nuclear layer; OS, outer segment. [file media-7.tif]
