# Supplementary material for: Mapping protein distribution in the canine photoreceptor sensory cilium and calyceal processes by ultrastructure expansion microscopy
Source: bioRxiv. 2024 Sep 22:2024.06.27.600953. Preprint. [Version 3] doi: 10.1101/2024.06.27.600953 (PMC11230445; doi:10.1101/2024.06.27.600953)
Supplement: Supplement 8 [file media-8.pdf]

Supplementary Table 1. Normal adult canines used in this study.

Dog ID	Eye	Sex	DOB	DOD	Age (months)	Storage at -80°C
**Fresh retina fixed using short duration**						
1180608	OS	M	4-May-2023	14-Sep-2023	4	n/a
RC724	OD	M	9-Jul-2022	28-Sep-2023	14	n/a
RC724	OS	M	9-Jul-2022	28-Sep-2023	14	n/a
**Fresh retina fixed using long duration**						
1180560	OD	M	4-May-2023	12-Sep-2023	4	n/a
1180560	OS	M	4-May-2023	12-Sep-2023	4	n/a
**Frozen archival retina**						
GI97	OD	F	15-Apr-2010	29-Nov-2010	7	> 12 years
GI97	OS	F	15-Apr-2010	29-Nov-2010	7	> 12 years
RC667	OD	F	17-Mar-2010	3-Nov-2010	7	> 12 years
RC667	OS	F	17-Mar-2010	3-Nov-2010	7	> 12 years

DOB, day of birth; DOD, day of death; F, female; M, male; OD, right eye; OS, left eye.

Supplementary Table 2. Reagents and materials used in this study.

Product	Supplier	Reference
**Tissue processing and storage**		
20% paraformaldehyde solution	Electron microscopy sciences	15713-S
Sterile Disposable Biopsy Punch 5mm	Sklar	96-1120
Sucrose	Fiser chemical	S2-500
Tissue-Tek O.C.T. Compound	Sakura finetek	4583
Tissue Embedding Disposable Molds	EBSciences	H1513
**U-ExM**		
14-mm microwell/35-mm petri dish	MatTek	P35G-1.5-14-C
12 mm Circular Cover Glasses	Fisher Scientific	12541001
Acrylamide	Sigma-Aldrich	A4058
Ammonium Persulfate (APS)	Bio-Rad	1610700
Formaldehyde solution	Sigma-Aldrich	F8775
Nuclease-Free Water	Invitrogen	AM9937
ImmEdge Pen	Vector laboratories	H-4000
N, N′-methylenbisacrylamide (BIS)	Sigma-Aldrich	M1533
Phosphate Buffered Saline (PBS), 10x	Bio-Rad	161-0780
Poly-D-Lysine	Gibco	A3890401
Sodium Acrylate (SA)	Sigma-Aldrich	408220
Sodium Chloride (NaCl)	Fisher Chemical	S271-3
Sodium Dodecyl Sulfate (SDS)	Fisher Chemical	BP166-500
Tetramethylethylenediamine (TEMED)	Bio-Rad	161-0800
Tris Base	Fisher Chemical	BP152-5
**Immunohistochemistry**		
Bovine Serum Albumin	Sigma-Aldrich	A7906
D-PBS with calcium & magnesium	Corning	21-030-CM
Gelatin from cold water fish skin	Sigma-Aldrich	G7765
Hoechst 33342	Thermo Scientific	62249
Pepsin Reagent, Antigen Retriever	Sigma-Aldrich	R2283
Sodium Azide	Sigma-Aldrich	S8032
Triton X-100	Fisher Scientific	BP151
Tween20	Bio-Rad	170-6531

Supplementary Table 3. Primary antibodies used in this study.

Target	Host species	Supplier	Reference	Dilution
Acetylated α-tubulin	Mouse monoclonal IgG2b	Sigma-Aldrich	T7451	1/1000
Acetylated α-tubulin	Rabbit monoclonal IgG	abcam	ab179484	1/1000
α-tubulin (non-acetylated)	Rabbit monoclonal IgG	abcam	ab18251	1/1000
ARL13B	Rabbit polyclonal IgG	Proteintech	17711-1-AP	1/200
ARL13B (clone N295B/66)	Mouse monoclonal IgG2a	abcam	ab136648	1/200
β-actin	Rabbit polyclonal IgG	abcam	ab8227	1/500
Blue-opsin	Rabbit polyclonal IgG	Sigma-Aldrich	AB5407	1/500
CCDC66	Rabbit polyclonal IgG	Invitrogen	PA5-60642	1/200
*CCDC66	Rabbit polyclonal IgG	Invitrogen	PA5-46125	1/200
CCDC66	Rabbit polyclonal IgG	Santa Cruz Biotechnology	sc-102418	1/200
Centrin	Mouse monoclonal IgG2aκ	Sigma-Aldrich	04-1624	1/200
CEP164	Rabbit polyclonal IgG	Proteintech	22227-1-AP	1/200
CEP290	Rabbit polyclonal IgG	Proteintech	22490-1-AP	1/200
Cone arrestin	Goat polyclonal IgG	Custom made (Beltran Lab)	ref. 63	1/400
Espin	Mouse monoclonal IgG1κ	Santa Cruz Biotechnology	sc-515657	1/200
FAM161A	Rabbit polyclonal IgG	Invitrogen	PA5-56935	1/100
γ-tubulin	Mouse monoclonal IgG1	Sigma-Aldrich	T6557	1/1000
Glutamylation (GT335)	Mouse monoclonal IgG1κ	AdipoGen	AG-20B-0020	1/1000
IFT57	Rabbit polyclonal IgG	Novus	NBP1-32932	1/200
KIF3A	Rabbit polyclonal IgG	abcam	ab11259	1/100
LCA5	Rabbit polyclonal IgG	Proteintech	19333-1-AP	1/200
MAP9	Rabbit polyclonal IgG	Proteintech	26078-1-AP	1/200
NPHP5	Rabbit polyclonal IgG	Proteintech	15747-1-AP	1/200
PCDH15	Sheep polyclonal IgG	R&D systems	AF6729	1/300
POC5	Rabbit polyclonal IgG	Bethyl	A303-341A	1/200
Red/Green-opsin	Rabbit polyclonal IgG	Sigma-Aldrich	AB5405	1/500
Rhodopsin	Mouse monoclonal IgG1	Sigma-Aldrich	MAB5316	1/1000
Rhodopsin	Rabbit polyclonal IgG	Sigma-Aldrich	AB9279	1/1000
Rootletin	Human monoclonal IgG	AbD Serotec	HCA009	1/200
RP1	Chicken polyclonal IgY	Custom made (Liu Lab)	ref. 64	1/200
RPGR	Rabbit polyclonal IgG	Proteintech	16891-1-AP	1/200
RPGRIP1	Rabbit polyclonal IgG	Sigma-Aldrich	HPA042955	1/200
SDCCAG8	Rabbit polyclonal IgG	Novus	NBP2-13288	1/200
SPATA7	Rabbit polyclonal IgG	Proteintech	12020-1-AP	1/200
* VLGR1	Rabbit polyclonal IgG	Atlas Antibodies	HPA067503	1/100
Whirlin	Rabbit polyclonal IgG	Proteintech	25881-1-AP	1/200

* No unique signals could be detected in the U-ExM samples.

Supplementary Table 4. Secondary antibodies and lectin fluorescent conjugates used in this study.

Target	Host species	Alexa Fluor	Supplier	Reference
Chicken IgY (H+L)	Goat polyclonal IgG	488	Invitrogen	A-11039
Mouse IgG (H+L)	Donkey polyclonal IgG	Plus 488	Invitrogen	A-32766
Mouse IgG2b	Goat polyclonal IgG	488	Invitrogen	A-21141
Sheep IgG (H+L)	Donkey polyclonal IgG	488	Invitrogen	A-11015
Human IgG (H+L)	Goat polyclonal IgG	568	Invitrogen	A-21090
Mouse IgG1	Goat polyclonal IgG	568	Invitrogen	A-21124
Mouse IgG2a	Goat polyclonal IgG	568	Invitrogen	A-21134
Mouse IgG (H+L)	Donkey polyclonal IgG	568	Invitrogen	A-10037
Rabbit IgG (H+L)	Donkey polyclonal IgG	568	Invitrogen	A-10042
Goat IgG (H+L)	Donkey polyclonal IgG	Plus 647	Invitrogen	A-32849
Mouse IgG1	Goat polyclonal IgG	647	Invitrogen	A-21240
Rabbit IgG (H+L)	Goat polyclonal IgG	647	Invitrogen	A-21246
Wheat Germ Agglutinin (WGA)	-	Fluorescein	Vector Laboratories	FL-1201

All secondary antibodies were used at a 1/1000 dilution for non-expanded IHC and 1/500 for U-ExM staining protocols.

Supplementary Table 5. Summary of immunohistological analysis for CCDC66 and ARL13B from previous publications and the current study.

CCDC66

**Retinal tissues**	Mouse, Dog, Human	Mouse	Dog	Dog		
**Antibodies**	Cat. #sc-102418		Cat. #PA5-46125	Cat. #PA5-60642	Cat. #PA5-46125	Cat. #sc-102418
**Epitopes**	Human CCDC66 (*an internal region)		Human CCDC66 (AA 45-94)	Human CCDC66 (AA 355-449)	Human CCDC66 (AA 45-94)	Human CCDC66 (*an internal region)
**Methods**	IHC	IHC	IHC	1) IHC (U-ExM)<br>2) IHC (no-expansion)	1) IHC (U-ExM)<br>2) IHC (no-expansion)	1) IHC (U-ExM)<br>2) IHC (no-expansion)
**Distribution in photoreceptors**	IS (**OS)	OS	OS	1) **Ciliary axoneme**<br>2) IS + CC	1) No detection<br>2) OS	1) **Ciliary axoneme**<br>2) No detection
**References**	Dekomien G et al. 2010 (ref. 29)	Gerding WM et al. 2011 (ref. 30)	Murgiano L et al. 2020 (ref. 31)	Current study		

* Immunogen sequence not provided.
** Faint immunoreactivity in the OS.

ARL13B

**Retinal tissues**	Mouse	Mouse	Mouse	Dog	
**Antibodies**	Custom made	1) Cat. #17711-1-AP<br>2) Clone N295B/66, Cat. #75-287	Clone N295B/66, Cat. #75-287	Cat. #17711-1-AP	Clone N295B/66, Cat. #ab136648
**Epitopes**	Mouse ARL13B (AA 208–428)	1) Human ARL13B (AA 1-321)<br>2) Mouse ARL13B (AA 208-427)	Mouse ARL13B (AA 208-427)	Human ARL13B (AA 1-321)	Mouse ARL13B (AA 208-427)
**Methods**	IHC	IHC	IHC	1) IHC (U-ExM)<br>2) IHC (no-expansion)	1) IHC (U-ExM)<br>2) IHC (no-expansion)
**Distribution in photoreceptors**	IS	OS (***IS)	OS	1) IS (Ciliary rootret)<br>2) IS (Ciliary rootret)	1) OS<br>2) OS
**References**	Kim YK et al. 2013 (ref. 51)	Hanke-Gogokhia C et al. 2017 (ref. 53)	Dilan TL et al. 2019 (ref. 52)	Current study	

*** Partial and limited distribution in the IS.
